# A rare case of residual root myiasis caused by *Clogmia albipunctata* larvae (*Diptera: Psychodidae*)

**DOI:** 10.1186/s12879-022-07325-2

**Published:** 2022-04-13

**Authors:** Juan Chen, Jinrui Liu, Yun Liu, Yingjie Liu

**Affiliations:** 1 Department of Pathogenic Biology, SchoolofBasicMedicalSciences, Henan University, Kaifeng, China; 2 Department of Gynecology and Obstetrics, Kaifeng New District Maternal and Child Health Care Hospital, Kaifeng, China; 3 Grade 2021, The Special Class for the Gifted Young, Xi’an Jiaotong University, Xi’an, China; 4 Department of Inorganic Chemistry, College of Chemistry and Chemical Engineering, Henan University, Kaifeng, China

**Keywords:** Myiasis, Residual root, *Clogmia albipunctata*, Larvae, Case report

## Abstract

**Background:**

Dental injury caused by caries and trauma is the main cause of residual roots. Food trapped in the residual roots is difficult to clean. If the residual roots are not treated and cared for in time, flies can take advantage as soon as hygiene slips. Here, we present a rare case of human residual root myiasis caused by *Clogmia albipunctata* larvae, never previously reported.

**Case presentation:**

A 26-year-old lady found two active, living larvae in her mouth while brushing her teeth. She did not present with fever, pain and any uncomfortable oral feeling. The intraoral examination revealed the right second mandibular molar was severely damaged as a result of caries, leaving a residual tooth root. The mucosa above it was mildly erythematous and edematous. No larvae and no inflamed gums were observed in her mouth. When normal saline was used to flush the area of the residual root with a syringe, four larvae appeared from the residual root. The larvae were observed by naked eye and under a light microscope. They were identified as the mature stage larvae of *Clogmia albipunctata*. Because the patient was in lactation, medication was not recommended. Treatment included the removal of all visible larvae followed by flushing the residual root with normal saline three times a day. The patient was followed-up weekly for one month. No more larvae were found and the erythematous and edematous mucosa healed completely.

**Conclusions:**

The existence of a residual root can result in residual root myiasis. Myiasis caused by *Clogmia albipunctata* larvae or other fly larvae should be considered in cases of residual root infection.

## Background

Human myiasis is defined as the infestation of the tissue of living human with dipterous larvae. It occurs worldwide [1, 2]. In humans, the sites most commonly affected are skin, nose, ears, eyes, anus, vagina, and oral cavity [2, 3]. Oral myiasis of humans is a pathology associated with a medical condition, poor oral hygiene, mouth breathing, and incompetent lip [4]. Here, we report a rare case of human oral myiasis caused by *Clogmia albipunctata* larvae, never previously reported.

## Case presentation

A 26-year-old woman was referred from Kaifeng New District Maternal and Child Health Care Hospital to the Department of Pathogenic Biology, Medical College of Henan University with the chief complaint being that she had found two living, active fly larvae in her mouth while brushing her teeth in the morning.

**Fig. 1:**
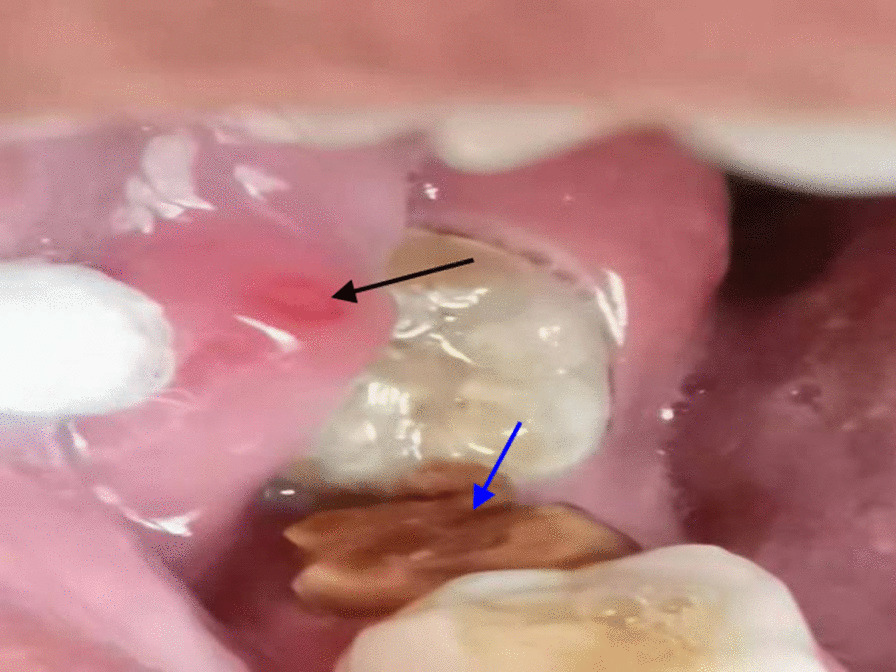
The residual root (blue arrow) and the mildly erythematous edematous mucosa (black arrow)

The woman was a civil servant living in the suburb of Kaifeng, Henan Province, China. She did not report fever, pain or any uncomfortable oral feeling. However, she appeared anxious and frightened. The initial intraoral examination revealed no larvae and no inflamed gums were observed in her mouth. Two years ago, her right second mandibular molar was severely damaged as a result of caries, leaving a residual tooth root. The patient never treated and cared of the residual tooth root except for brushing her teeth once a day. The mucosa above the residual root was mildly erythematous and edematous and no bleeding was present (Fig. 1).

When normal saline was used to flush the area of the residual root with a syringe, a total of four larvae mixed with a little of trapped, decomposed food were flushed out of the residual root. The larvae were removed quickly and gently using forceps. They wriggled around actively in water and crawled quickly on a solid surface.

Because the patient was in lactation, medication was not recommended. Treatment included the removal of all visible larvae followed by flushing the residual root with normal saline. She was advised to flush the residual root with normal saline three times a day before brushing her teeth. We advised her to refer to dentist to treat the residual root as soon as possible. We also advised her to clean the room to eliminate the breeding environment of the flies and spray with insecticides to exterminate the flies. The patient was followed-up weekly for one month. No more larvae were found and the erythematous and edematous mucosa healed completely.

**Fig. 2:**
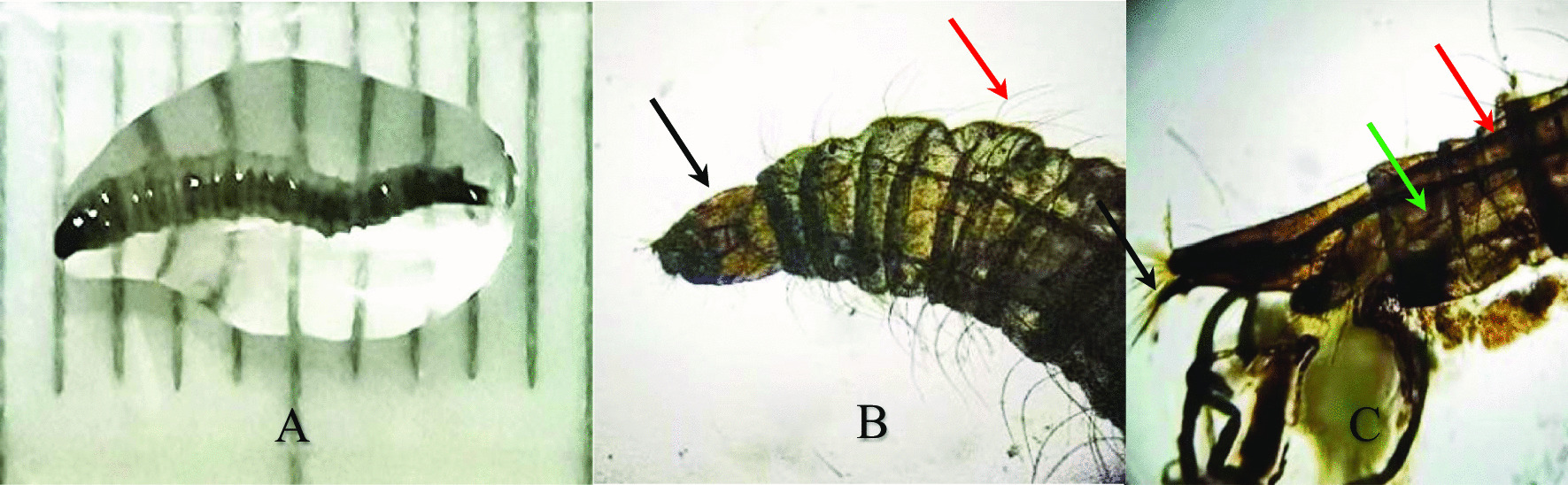
*Clogmia albipunctata* larva. **A** Full-larva. **B** The ovoid head (black arrow) and the thoracic segments and a part of abdominal segments with long dark backwardly directed filiform setae (red arrow, 40×). **C** Caudal part showing dorsal plates (green arrow), 2 internal breathing tubes extending along the length of the body (red arrow) and end anal processes with a tuft of hairs at the end (black arrow, 40×)

The larvae were observed by naked eye and under a light microscope (Motic BA210, MOTIC CHINA GROUP CO., LTD.). The larvae were cylindrical and about 7–8 mm long and 1 mm wide. They were grayish dorsally and white ventrally, while the ovoid shaped head and the cone-shape tail were dark brown (Fig. 2A).The dorsal surface of the body segments were covered with 26 saddle-shaped dark chitinous plates. The mouthparts were of the chewing type. The body was densely covered with long black backward projecting setae dorsally and laterally (Fig. 2B, red arrow). Two internal breathing tubes appear extending along the length of the body starting at a pair of anterior spiracles on the prothorax and ending with a pair of posterior spiracles, at the tip of the terminal segment. Caudally, the siphon was cone-shape. There were two dorsal anal processes and two ventral anal processes with a tuft of hairs at the end (Fig. 2C, black arrow).

From previous morphological characters and comparing them to literatures [5–8], the larvae were identified as the mature stage larvae of *Clogmia albipunctata* (Diptera: Psychodidae)*.*

## Discussion and conclusions

Oral myiasis of humans is associated with poor oral hygiene, alcoholism, senility, mental debility, mouth breathing [9], incompetent lip, cerebral palsy [10], severe halitosis, suppurating lesions, gingival disease and trauma [11]. Poor oral hygiene is among the more important risk factors in oral myiasis. After careful taking of history from the patient, we learned she had the habit of sleeping with her mouth open. Because she was in lactation, there were many fruits and snacks in the bedroom which attracted some small flies. It was concluded that the patient did not look after her oral hygiene. Food trapped in the residual root is difficult to clean. While the patient slept with her mouth open, the smell of the trapped, decomposed food in the residual root attracted one or more flies to lay eggs in the residual root.

The main species reported to cause oral myiasis are *Cochliomyia hominivorax*, *Chrysomya bezziana*, *Musca domestica*, *Sarcophaga* species, *Luciliasericata*, *Lucilia cuprina*, *Musca nebulo*, *Oestrus ovis*, *Calliphoridae*, *Dermatobia hominis*, *Hypoderma bovis*, *Hypoderma tarandi* and *Wohlfahrtia magnifica *[2]. Larvae of *Clogmia albipunctata* had been reported to cause human nasopharyngeal myiasis [12], intestinal myiasis [6, 8] and urinary myiasis [5, 7]. However, the case of human residual root myiasis caused by *Clogmia albipunctata* larvae had never been reported.

*Clogmia albipunctata* is a primitive Nematoceran of the family Psychodidae, subfamily Psychodinae and cosmopolitan in distribution. The adult flies can survive and spread outdoors during the temperate seasons. *Clogmia albipunctata* can elicit inhalant allergy as a result of inhaling fragments of their disintegrated body parts and can play a significant role as a potential mechanical vector of pathogens [13]. Their larvae are coprophagous and saprophagous. They feed on decaying organic matter and vertebrate feces. They are present in moist places such as bathrooms and toilets [14]. Because they are non-biting, tiny and quiet, most people do not notice them. The treatments of myiasis include manual removal of larvae and debridement, application of antibiotic therapy, asphyxiating substances and ivermectin. Because the patient was in lactation, we used just the treatment of manual removal of larvae and flushing the area of the residual root with normal saline.

In summary, good oral hygiene is important in all circumstances and residual roots should be treated and cared for in time until it is fully healed, as flies can take advantage as soon as hygiene slips. The existence of a residual root can result in residual root myiasis. Myiasis caused by *Clogmia albipunctata* larvae or other fly larvae should be considered in cases of residual root infection.

## Data Availability

All data discussed in the manuscript are included within this published article.
